# The Physiological Action of the Sulphates of Potass, Soda, and Magnesia When Injected in the Blood

**Published:** 1869-05

**Authors:** F. Jolyet


### THE PHYSIOLOGICAL ACTION OF THE SULPHATES OF POTASS, SODA, AND MAGNESIA WHEN INJECTED IN THE BLOOD.

By MM. F. JOLYET AND CAHOURS.

Translated for the *Medical Examiner*.

We propose in this article to demonstrate—

1st. That the neutral salts (sulphate of soda and sulphate of magnesia) which are daily employed as purgatives in the intestine do not produce purgation, when injected in the veins.

2d. That these same injections enable us to distinguish the sulphates of potass, soda, and magnesia by their poisonous properties, and their physiological effects.

Physiologists frequently have injected the different salts of potassium and sodium in the blood, for the more special purpose of distinguishing these two alkaline metals by their poisonous properties. The most delicate experiments on this subject have been made by M. Grandeau. (*Vide Journal de l'Anatomie et de la Physiologie, etc.*, by M. Ch. Robin, 1864: Experiments upon the Physiological Action of the Salts of Potassium, of Sodium, and of Rubidium.) They show—

1st. That the salts of soda can be introduced in the circulation without producing accident, and that very large doses do not lead to fatal results.

2d. That the salts of potass, injected in the blood, are eminently poisonous, and that very minute portions suffice to produce a frightful death.

The experiments of M. Cl. Bernard have taught that the salts of potass direct their action upon the muscular tissue, and that death, caused by the injection of these salts in the blood, is due to the sudden arrest of the heart, before the cessation of the respiratory movements. In his investigations upon the physiological action of the salts of potassium, and of sodium, P. Guttman (*Berliner Klinische Wochenschrift*, 1865, Numbers

3435 and 3436,) examined more closely than any one had before him the mode of action of these compounds. According to his observations, all the salts of potassium (with the exception of bromide and iodide, and some salts, the acid of which is poisonous in itself) have an equally toxical property, and all act directly upon the heart. They diminish, at first, the force and frequency of its pulsations, and they effect its arrest by paralysis of the muscular action of the organ, which, once at rest, does not react under electrical excitement. The greatest difference between the physiological action of the salts of potassium and the salts of sodium is, that the latter exercise no influence upon the heart—as Blake has already observed (*Edinburgh Medical Journal*, 1839).

Upon these two points—poisonous properties, and intimate action of the salts of potass—our experiments with the sulphate of potass, alone, confirm the data already given.

What the action of the salts of magnesium, and, particularly, of the sulphate of magnesia, may be, has not been investigated very thoroughly, at least to our knowledge; and we should hesitate to accept the opinion of M. Rabuteau (*Etude experimentale sur lès effets physiologiques des fluorures et composes metalliques en general; These de Paris:* 1867), who, after one experiment with the chloride of magnesium, concluded that this metal is as entirely harmless as sodium.

SULPHATE OF POTASS.

The experiments we are about to report are designed to show the action which the sulphate of potass exercises upon the heart and the muscles. To evince clearly the action upon the heart, it is necessary to experiment upon an animal, withdrawn from all causes that can modify the rythm, and the frequency of the pulsations of this organ. To accomplish that, we have employed the method of double poisoning. We have made our experiments upon dogs subjected to the influence of curare, until the excitability of the motive organs by electricity is completely destroyed. By this procedure, and maintaining an artificial respiration, we can expose the heart to view, and observe, directly, the troubles arising from the introduction of a foreign substance in the circulation.

Example I. July 8th, 1868. Poisoned a small-sized bitch, by injecting under the skin some centigrammes of curare, in solution. After a short time, signs of poisoning manifested themselves. Artificial respiration was maintained for an hour. At that time, the sciatic nerve laid bare was no longer excitable. The heart was exposed; the pulsations were very regular, but sufficiently frequent. (The two pneumogastrics were severed.) At 9 h. 55 m., two cubic centimètres of a solution of sulphate of potass of 10e (that is, 20 centigrammes of sulphate of potass) were injected carefully in the left crural vein.

The phenomenon which suddenly manifested itself in the region of the heart was a diminution, a kind of hesitancy, in the pulsations.

This diminution, which was only transient, was succeeded, almost immediately, by a greatly accelerated action; there were, as far as one could judge, a-third or a-half more strokes than before the injection.

Then came a time when the pulsations were disturbed. They presented an accelerated series, separated by moments of cessation—a series of vigorous throbbing, followed by feeble action. Then, the pulsations became less and less frequent, and were replaced by the partial and, as it were, vermicular contractions of the ventricle, which became distended, in a measure, by the blood, which it could no longer expel. 10 h. 1 m. Complete cessation of the heart in diastole. The blood of the heart, collected in a saucer, coagulated with great rapidity.

This experiment has been repeated three times with identical results. With dogs of small size it was sufficient to introduce two and three cubic centimètres of the solution of sulphate of potass to produce the trouble of the heart, and its complete arrest, in some minutes. In another series of experiments, we have practiced injecting sulphate of potass in the blood of frogs.

The following is the process at which we have arrived:—

We make the injection in the arterial system. In order to do that, we expose the terminal part of the aorta and its bifurcation into the two iliac arteries; and it is in one of these arteries that we introduce a fine canula and make the injection.

The canula of the syringe is introduced in the artery, very near the aorta, in such a manner that when the liquid is injected, by the eighth of a drop, from the syringe of Parvas, that which passes immediately into the artery of the opposite side diffuses itself throughout the corresponding posterior member, and is returned immediately to the heart by the venous system.

This method has the advantage, at the same time, of isolating one of the members of the animal (the posterior member, in the artery of which the injection is made) from the action of the substance.

EXAMPLE II. 2 h. 26 m. 15 centigrammes of the solution of sulphate of potass, of 10e, are injected in the left iliac artery of a green frog, whose heart beats 70 times in a minute.

While the injection is made very carefully, slight fibrile contractions are observed in the muscles of the thigh, then in those of the calf, and some trembling of the toes of the right side. These contractions, which impart a little rigidity to the member, cease in about two minutes. The limb, released and extended, does not retract.

2 h. 30 m. The heart is observed; 20 pulsations a minute. The ventricle remains a comparatively long time in diastole. Its contraction is unequal; some parts of the ventricle close, while others do not empty themselves. The auricle contracts with difficulty and irregularity.

The frog, being released, executes some energetic movements of the right hind foot; the left remains extended and paralyzed. Feebleness of the anterior parts, which are somewhat depressed, sensibility preserved throughout.

2 h. 38 m. 50 pulsations of the heart; more regular; the auricle contracts with more power; repose of the heart more or less prolonged from time to time. Motion begins to return in the right posterior member; and the thigh flexes itself gently upon the body.

2 h. 50 m. 60 beats of the heart; full and regular.

4 h. 10 m. Normal condition of the frog:—right posterior member very active; left posterior member (vessels ligated) grows paralyzed.

Example III. The arteries and lumbar nerves of a large frog are severed; and, at 12 h. 52 m., 20 centigrammes of the solution of sulphate of potass are injected in the left iliac artery. During the process of injection, the fibrile contractions, which, at that time, usually manifest themselves in the right foot, are not apparent.

1 h. The lumbar nerves of the right side are excited by employing the forceps of Pulvermacher. No contractions in the muscles of the foot. The same excitant directed against the muscles of the foot fails equally to provoke any contraction.

On the contrary, contractions are plainly evident when the forceps are applied to the muscles of the left foot (vessels ligated). Besides, the animal executes some energetic movements with this foot.

1 h. 5 m. and 1 h. 10 m. Same results—non-excitability of the muscles on the right; excitability on the left.

1 h. 20 m. The muscles of the right thigh begin to contract. This is shown likewise by electricizing the lumbar nerves. The part is nevertheless paralyzed.

1 h. 35 m. Spontaneous movements in the right hind foot, which has regained its energy, in a great measure.

Among the frogs, the difficulties arising in the heart's action are only transient, and have never occasioned the arrest of that organ. The reason is, the doses of sulphate of potass (1 to 2 centigrammes) are too small.

The same hesitation, the same irregularities in the cardiac pulsations, are observed, either when the frogs are well or previously poisoned by curare. Only one phenomenon is lacking, in every instance, among the frogs under the influence of curare, that is, the muscular fibrile contraction, that, in the healthy frog, almost invariably manifests itself in the posterior member, where the liquid of the injection passes immediately.

### SULPHATE OF SODA.

The almost absolute harmlessness of injecting the salts of soda in the blood enables us to determine whether the sulphate of soda, injected in the veins, purges, as when given by the intestine.

*A priori*, and, by analogy, it seems to be thus. It appears perfectly natural that the same as an emetic injected in the veins produces vomiting, Glauber salts should purge. It is undoubtedly upon these data, only, that cathartic properties have been attributed to the sulphate of soda injected in the blood.

EXAMPLE IV. July 27th, 1868: 11 A.M. 12 grammes of sulphate of soda dissolved in 40 grammes of water were injected in the crural veins of a dog weighing 10 kilogrammes. Immediately after the injection, and even before it was terminated, the respiration of the animal growing embarrassed became difficult and wheezing. This trouble continued a little time, and, presently, respiration was natural. The animal released and placed on the ground went about the laboratory, appearing a little more feeble in the posterior region, and after a time laid down. He was seized with a slight trembling or shivering, which continued nearly five hours.

At six o'clock in the evening, that is, after a period of seven hours, the animal was observed and had no evacuation, neither solid nor liquid. He had remained all this time lying in the same position.

The next day (July 28th), at 8 A.M., the animal appeared quite recovered from the injection: from the depression which at first ensued, he has become sportive and fawning. There was in the kennel only one scanty evacuation, half liquid, half solid, bilious, of recent date.

July 29th. Solid evacuations.

July 31st. The animal has had quite profuse hemorrhage in the night. The wound in the thigh still bleeds easily. No reunion by first intention of the wound, the edges of which are gaping and scarcely swollen.

This experiment repeated several times shows:—

1st. That the sulphate of soda, when injected in the veins, does not purge.

2d. That by the diminution of the coagulability and plasticity which it occasions in the blood, the salt of soda disposes to hemorrhages, and retards the work of cicatrization.

SULPHATE OF MAGNESIA.

EXAMPLE V. September 17, 1868. 15 cubic centimètres of a solution, 20 parts sulphate magnesia to 100 parts water, were injected in the right crural vein of a vigorous dog, weighing 8 kilogrammes. At 2 h. 16 m., during the injection, the animal was restive, its respiration accelerated, then became feeble, and, presently, was carried on by short and abrupt contractions of the diaphragm.

2 h. 22 m. New injection of 15 cubic centimètres of the solution. Complete arrest of respiration. Artificial respiration maintained by means of bellows.

No reflex action of the eyes, upon touching the cornea. No movements of the foot or tail, when pinched severely. The heart beats very regularly.

2 h. 30 m. The left sciatic nerve was galvanized by means of the apparatus of Legendre and Morin: very feeble contraction of the muscles of the member. The muscles under direct excitement contracted well. No reflex movements of the eyes.

2 h. 35 m. Galvanization of the sciatic nerve; no movement of the foot. Muscles excitable; no reflex action; pupils largely dilated.

2 h. 40 m. Spontaneous respiration commenced in the diaphragm; but weak and insufficient.

2 h. 45. Sciatic nerve became slightly excitable. Abdominal respiration stronger and more regular (artificial respiration discontinued.) No reflex action of the eyes or feet, when pinched hard.

2 h. 55 m. Feeble effort to close the eyelids, upon irritation of the cornea.

3 h. Galvanization of the sciatic nerve; decided motion in the foot. Strong and full respiration, which indicated that the animal perceived the uncomfortable sensation, caused by excitement of the nerve.

3 h. 20 m. Voluntary movement of the head and hind feet. Reflex action of the eyes partially returned. Pupils less dilated.

3 h. 30 m. General tremor commenced in the anterior parts, and extended itself to the hind feet, in proportion as motion returned there. It ceased about 4 o'clock: at that time the animal had regained all its movements, but its gait was still feeble and uncertain.

September 18th. Two bilious liquid evacuations in the night.

September 22d. The animal had recovered partially. There was no immediate reunion of the wounds in the neck and thigh; the edges were gaping, and nearly free from tumefaction. On pressure of the wound, a bloody serum exuded.

September 25th. The wounds commenced to suppurate, especially that of the neck, which was in a more advanced state of cicatrization.

Example VI. Frog. 25 centigrammes of a solution, one part sulphate of magnesia to three parts water, was injected in the left iliac artery, at 6 h. 55 m. Injection finished at 7 h. 5 m.

7 h. 15 m. Complete paralysis of voluntary and reflex action, save in the left posterior member, preserved by the ligation of its vessels. Lumbar nerves of the right side not excited by the forceps of Pulvermacher. Muscles excited directly. Regular action of the heart.

The next day, the frog has regained full power of motion.

One fact results from our experiments with the sulphate of magnesia, it is: that injections of this salt in the blood are poisonous. We cannot admit, then, with M. Rabuteau, that the salts of magnesium are as harmless as the salts of sodium. In our experiments with the sulphate of soda, we have injected, always, from 10 to 15 grammes, and sometimes 20 grammes of this salt, without producing death. On the contrary, 2 to 6 grammes of sulphate of magnesia, according to the size of the animals, have invariably sufficed to occasion an almost frightful death.

This first point established, let us seek to ascertain the mode of intimate action of the sulphate of magnesia. In consideration of these facts, only, paralysis of voluntary and reflex action, bordering upon loss of the excitability of the motive nerves, with preservation of the action of the muscles, which follow the introduction of the substance in the blood, among dogs; the same effects, with greater preservation of the sensibility and movements of the isolated member, among frogs. One cannot refrain from comparing the action of sulphate of magnesia with the characteristic action of curare, and the poisons of the motive nerves. Without doubt, the alteration that the sulphate of magnesia originates in the blood should be taken into consideration; but this alteration would not give an explanation of the phenomena which immediately follow the injection. The diminution of the coagulability and plasticity of the blood can only, as with sulphate of soda, account for the later phenomena, that is, the consecutive hemorrhages and delay occasioned in the cicatrization of the wounds.

Experiment V. shows, finally, that if the sulphate of magnesia injected in the blood sometimes produces liquid and bilious evacuations, properly speaking, it does not purge, as when given in the intestine.*

In the course of experiments made in April, 1867, to study the toxical action of different metalic salts, I introduced a very small quantity of crystallized sulphate of magnesia under the skin of a frog's back. The animal became gradually enfeebled: at the end of an hour, there was no movement, either spontaneous or reflex, in the different parts of the body. It was the same when sulphate of potass was introduced, hypodermically; but sulphate of soda had no analogous effect. The chlorhydrate of ammonia, on the contrary, occasions, also, an abolition of spontaneous and reflex action, excepting that of the heart, but after having produced a more or less transient tetanic condition. In many cases, the effects of these salts have been dissipated completely at the expiration of a few hours.—[A. VULPIAN.

*Since this article was sent to the press, we have learned that M. Rabuteau and M. A. Moreau have each arrived at the same result as ourselves, relative to the non-carthartic action of sulphate of soda injected in the blood.

